# The application of the loop-mediated isothermal amplification (LAMP) method for diagnosing *Enterococcus hirae*-associated endocarditis outbreaks in chickens

**DOI:** 10.1186/s12866-019-1420-z

**Published:** 2019-02-21

**Authors:** Beata Dolka, Agata Anna Cisek, Piotr Szeleszczuk

**Affiliations:** 1 Department of Pathology and Veterinary Diagnostics, Faculty of Veterinary Medicine, Warsaw University of Life Sciences, Nowoursynowska 159c, 02-776 Warsaw, Poland; 2 Department of Preclinical Sciences, Faculty of Veterinary Medicine, Warsaw University of Life Sciences, Ciszewskiego 8, 02-786 Warsaw, Poland

**Keywords:** Broiler chickens, Endocarditis, *Enterococcus hirae*, LAMP, qPCR, CFU

## Abstract

**Background:**

*Enterococcus hirae* is considered a part of the normal intestinal biota of several domestic animals, including poultry. However, this species is also associated with infective endocarditis in chickens, a disease that leads to unexpected deaths and serious economical losses. *Enterococcus hirae* is identified predominantly with the use of conventional bacteriological methods, biochemical tests and PCR. Rapid, sensitive and specific methods for detecting *E. hirae* in clinical samples are required in poultry production. The aim of this study was to use the Loop-Mediated Isothermal Amplification (LAMP) for the identification and quantification of *E. hirae* in heart samples from broiler chickens.

**Results:**

The specificity of the LAMP method was confirmed for 7 enterococcal strains and 3 non-enterococcal strains. *E. hirae* was detected in all of the 22 analyzed clinical bacterial isolates and in all of the 9 heart samples. Three sets of primers supported the detection of *E. hirae* with high sensitivity and specificity within one hour. The highest detection rate of a LAMP product was approximately 7 min for an *E. hirae* strain and 12 min for a positive heart sample. The detection limit for the *E. hirae* ATCC 10541 standard was 1.3 × 10^2^ CFU (43.4 fg) or 13.8 copies of the *E. hirae* genome equivalent per reaction. The reaction was 10-fold more sensitive than conventional species-specific PCR. The LAMP assay supported the determination of the *E. hirae* load in chicken hearts with endocarditis in field cases. The average number of *E. hirae* cells in hearts was 5.19 × 10^7^ CFU/g of tissue, and the average number of *E. hirae* genome equivalents in hearts was 5.51× 10^6^ copies/g of tissue. Bacterial counts were significantly higher in the LAMP assay than in the standard plate count.

**Conclusions:**

The LAMP assay is a useful diagnostic tool and an effective alternative to conventional methods for the detection of this enterococcal species. The *sod*A-based LAMP assay supported direct identification of *E. hirae* from pure cultures and heart samples without previous bacterial cultivation. This is the first study to apply the LAMP method for the purpose of diagnosing *E. hirae-*associated endocarditis in poultry.

## Background

*Enterococcus hirae* is one of the 58 known species of the genus *Enterococcus*. *E hirae* was first described as a new species in 1985 by Farrow and Collins in strains that had been previously referred to as *Enterococcus faecium*. These authors established the distinct taxonomic position of *E. hirae* as a species that is separate from *E. casseliflavus*, *E. durans* and *E. faecium* [1]. *Enterococcus hirae* is considered a part of normal intestinal biota and an opportunistic pathogen in birds and mammals [2–4]. In the group of enterococci that constitute normal poultry microbiota, *E. hirae* has been relatively rarely isolated from the intestines. Devriese et al. [5] demonstrated that *E. hirae* colonizes the small intestines of 3- to 4-week-old chickens, and – less frequently − 12-week-old chickens, but the bacterium was not isolated from the crop or the cecum. In chickens from tropical regions, *E. hirae* was detected in cecal and cloacal swabs only in birds older than 8 weeks [6].

According to the literature, *E. hirae* is the fourth (2.7%) most common *Enterococcus* species identified in poultry [7]. In a study where samples were composed of 97% hearts, *E. hirae* was detected in 4.6% of the samples (after *E. faecalis –* 74.7%, *E. faecium –* 10.1%, *E. gallinarum –* 5.5%). *Enterococcus hirae* was isolated mainly from laying hens (CL) (8.3%), turkeys (5.6%) and broilers (CB) (5.5%), but it was never detected in broiler breeder chickens (BB) [8]. According to other authors *E. hirae* was more prevalent in ducks (6.2%) than in CB (3.6%), BB (2.4%), CL (1.8%) and turkeys (0.7%) [7]. *Enterococcus hirae* was not detected in geese [7, 8]. The mean age of birds at the time of *E. hirae* isolation was approximately 3 days in ducks, 4 days in BB, 12–13 days in CB, 4 weeks in CL, and 6 weeks in turkeys [7]. *Enterococcus hirae* was not identified in chicks (1- to 5-day-old) in 9 diagnostically independent cases, but it was more frequently isolated from older chickens (5 days – 6 weeks) (48%) [9].

According to the original reports from 1985, *E. hirae* was associated with growth depression in young chickens [1]. Saikia et al. [6] observed that this species could be potentially pathogenic in chickens younger than 1 week. Devriese et al. [10] were the first to determine that *E. hirae* could cause septicemia and focal necrosis of the brain in 3- to 8-day-old chicks. Cases of *E. hirae* septicemia were also noted in 10 species of psittacine birds [11]. *Enterococcus hirae* can cause encephalomalacia in 1- to 2-week-old broilers and layers [10, 12]. The bacterium should be also included in differential diagnoses of diarrhea in chicks [13] and osteomyelitis in broiler chickens [14]. Since the 1990s, *E. hirae* has been recognized as an important etiological agent of bacterial endocarditis in chickens [15–17]. The prevalence of *E. hirae* in the microbiome of chicken hearts with endocarditis is unknown. *E. hirae* infections are characterized by fibrinous thrombotic lesions in atrioventricular (AV) valves, less often in the lungs, the external ischiadic artery (*arteria ischiadica externa*), liver and brain vessels. Velkers et al. [17] detected *Enterococci* in 54% of the examined hearts. The percentage of affected hearts was highest in 2- to 3-week-old (47%) and 3- to 4-week-old broiler chickens (46%). In another study, *E. hirae* was isolated from 42% of birds (20-days-old) [16]. Outbreaks of *E. hirae*-associated endocarditis can cause economic losses in poultry production. Mortality peaks in the second week of broiler grow-out. *E. hirae* endocarditis is responsible for 36% of broiler deaths in the grow-out period. Lameness is occasionally observed [17].

In young birds, *E. hirae* infections may lead to only a minor increase in mortality without specific clinical signs. Outbreaks are not always clearly defined and may remain unnoticed or may be attributed to poor chick quality [16]. For this reason, the etiological agent and the prevalence of the infection are often not identified. An accurate diagnosis of the infection is crucial to avoid unnecessary antibiotic use and to select the most appropriate therapy. In most cases, diagnosis is considerably delayed, which could explain the presence of severe cardiac lesions during necropsy. The present study highlights the importance of proper diagnosis of enterococcal infections in poultry.

*E. hirae* is a zoonotic pathogen, but it rarely causes infections in humans [2–4]. Most human cases involved bacteremia accompanied by severe illness, such as acute pyelonephritis, pancreatitis, cholangitis, severe urinary tract infections or spondylodiscitis [4, 18–20]. Three cases of human endocarditis caused by *E. hirae* have been reported to date [3, 21, 22].

Standard methods of enterococci identification rely on conventional culture-based approaches, including evaluations of colony morphology, Gram staining and analyses of biochemical properties [2]. Additional tests are required to identify *E. hirae* to species level. However, selected enterococci of avian origin are not always detected by commercially available automated identification systems. The problems associated with the phenotypic identification of *E. hirae* have prompted the development of more accurate molecular techniques. At present, *E. hirae* are identified with the use of PCR techniques based on the amplification of the *sod*A gene encoding superoxide dismutase (Mn) and gene sequencing [23]. The detection of *E. hirae* by conventional culture- and biochemical-based assays is time-consuming, laborious and requires several days. PCR assays are more rapid than conventional methods, but they require agarose gel electrophoresis; therefore, the identification of *E. hirae* can be completed in a few hours.

In 2002, a new nucleic acid amplification method, loop-mediated isothermal amplification (LAMP), was described by Notomi et al. [24]. LAMP is based on an autocycling strand-displacement reaction which uses specific DNA polymerase (such as *Bst*, *Bsm* or Gsp) and a set of 4 primers (F3, B3, FIP, BIP) complementary to the target gene. Additional two loop-creating primers may be used to improve amplification [25]. DNA is amplified under isothermal conditions with high specificity, sensitivity, efficiency and speed. The LAMP technique has revolutionized the detection of poultry pathogens, and it is a highly useful tool for the rapid detection of selected viruses, bacteria, fungi and protozoa [26–33]. However, there are no published reports on the application of the LAMP method for the identification of *E. hirae*. In this study, the LAMP assay has been used to detect and quantify *E. hirae* responsible for endocarditis in broiler chickens. The LAMP technique was evaluated using a panel of bacterial DNA and heart samples from field outbreaks in broiler flocks. The results were compared with the outcomes of standard PCR and culture-based methods.

## Methods

### Bacterial strains and heart samples

**Fig. 1:**
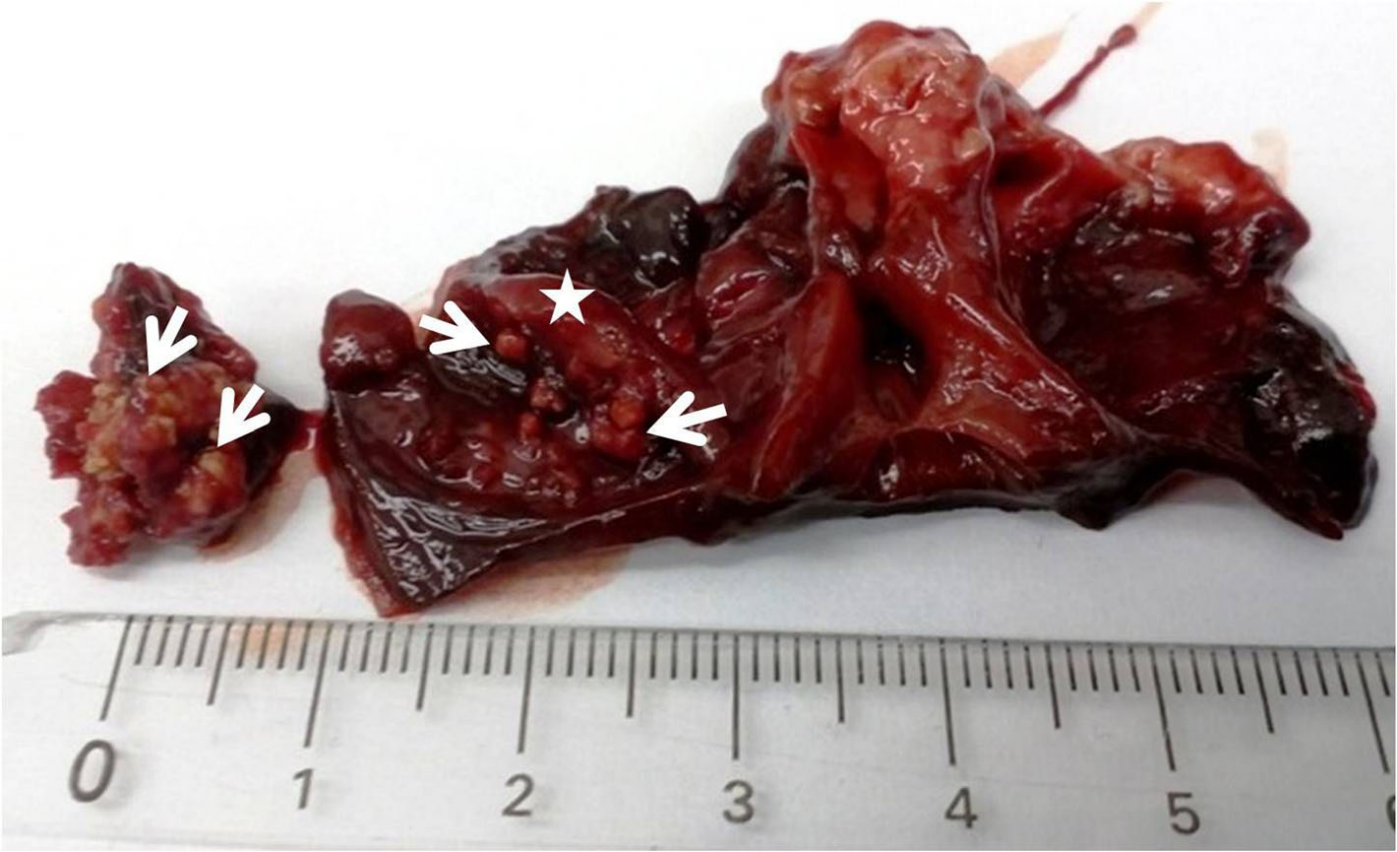
The chicken heart with severe lesions (arrows) on the right atrioventricular valve (asterisk) associated with *E. hirae* infection

A total of 22 field strains of *E. hirae* and 9 heart samples (tissue with lesions) from broiler chickens (CB) were analyzed. Each strain and heart represented a separate disease outbreak reported between 2011 and 2017 in Poland. All samples were obtained from the collection of the Division of Avian Diseases, Department of Pathology and Veterinary Diagnostics, Faculty of Veterinary Medicine of the Warsaw University of Life Sciences. Pursuant to the provisions of Polish and European Union law (Journal of Laws of 2015, item 266; Directive 2010/63/EU), the approval of the Animal Ethics Committee was not required for this study. Bacterial strains were isolated from heart samples suspected of enterococcal infection. Heart samples were collected during routine diagnostic necropsy of chickens from flocks suspected of *E. hirae*-associated endocarditis (Fig. 1). *E. hirae* was cultured from approximately 20–60% of hearts. Heart samples (valve fragments with heart blood) were plated onto Columbia agar supplemented with 5% sheep blood and onto Enterococcosel Agar plates containing esculin (Graso, Poland). They were incubated at 37 °C for 24 h in a CO_2_-enriched atmosphere. Bacterial isolates were initially characterized based on analyses of colony morphology (transparent grey to white colonies), Gram staining (Gram-positive) and catalase production (negative). The preliminary identification of *E. hirae* was carried out in the API Rapid ID 32 STREP system (bioMérieux France). Clinical strains were confirmed by sequencing (Genomed, Warsaw, Poland). The sequences of 6 randomly selected isolates were deposited in GenBank (MF356372 – MF356377).

### DNA isolation from bacterial strains

The DNA from clinical bacterial strains was extracted by the boiling lysis method. Single colonies were selected from Columbia agar (with 5% sheep blood) and suspended in 0.5 ml of sterile water. The cell suspension was held in a boiling water-bath for 10 min to lyse the cells; it was chilled on ice for several seconds and centrifuged for 5 min at 10000 rpm. A supernatant (1 μl) was used for LAMP and conventional PCR reactions.

### DNA isolation from heart samples

The DNA from the affected heart samples was extracted using the Sherlock AX kit (A&A Biotechnology, Gdańsk, Poland) with lysozyme*-*mutanolysin pretreatment. Briefly, 20 mg specimens of heart samples with visible lesions were transferred to 1.5 ml Eppendorf tubes, suspended in 300 μl of sterile water, 60 μl of lysozyme (10 mg/ml) and 5 μl of mutanolysin (10 U/μl). The solution was mixed in a vortex and incubated in the Thermomixer Compact (Eppendorf AG, Germany) at 37 °C for 15 min with shaking at 600 rpm. The solution was combined with 300 μl of lysis buffer L.1.4 and proteinase K (Sherlock AX, A&A Biotechnology, Gdańsk, Poland). It was vortexed (20 s) and incubated in the thermomixer at 50 °C with vigorous shaking at 1400 rpm for 60 min (until complete digestion). Samples were treated with 5 μl of RNAse (10 mg/ml) for 5 min at room temperature to remove residual bacterial RNA. Subsequently, the isolation was continued according to the first step of the Sherlock AX protocol. The final DNA pellet was dissolved in 20 μl of nuclease-free water (R0581, Thermo Fisher Scientific Inc., USA).

### LAMP assay

**Fig. 2:**
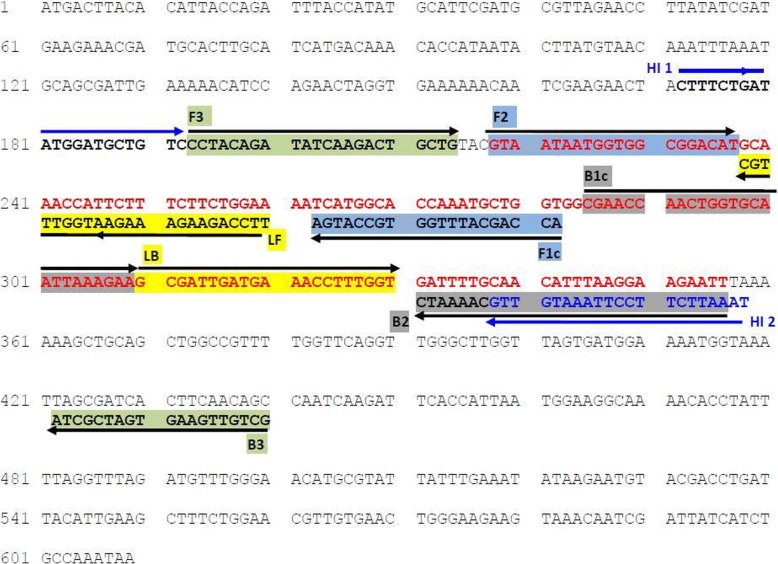
Location and sequences of primers used in the LAMP assays. The positions of the LAMP primers are shown relative to the *sod*A gene fragment of *Enterococcus hirae* (accession no. CP003504.1). Right and left arrows indicate sense and complementary sequences. Green boxes: indicate the outer primers F3 and B3 (product size 248 bp); Blue boxes: indicate forward inner primer FIP (F1c + F2); Grey boxes: indicate backward inner primer BIP (B1c + B2); Yellow boxes: indicate Loop primers LF and LB. Red font indicates the location and sequence of LAMP product (139 bp). Blue font indicates the location of species-specific primers used in standard PCR (product size 187 bp)

The nucleotide sequences of the primers are shown in Table 1 (Novazym Polska s.c., Poznań, Poland). The primers were designed using LAMP Designer (OptiGene Ltd., UK) based on NCBI sequences of the *sod*A gene in *E. hirae* (GenBank number: EU02133). The location of the primers within a gene fragment is shown in Fig. 2. The total reaction mixture (12.5 μl) consisted of: 7.5 μl of the Isothermal MasterMix (ISO-001 OptiGene Ltd. UK), 3 μl of the primer mix, 1 μl of nuclease-free water and 1 μl of DNA. The primer mix consisted of: 0.5 μl of F3, 0.5 μl of B3, 2 μl of FIP, 2 μl of BIP, 1 μl of LoopF and 1 μl of LoopR, at 10 pmol/μl each. The LAMP assay was performed with the Stratagene Mx3005P QPCR instrument (Agilent Technologies Inc., Santa Clara, CA, USA) with a melting curve analysis step. The mixture was incubated for 80 cycles of 65 °C for 30 s. Fluorescence was measured after each cycle in the FAM channel. For melting (dissociation) curve analysis, the temperature was increased gradually from 65 °C to 95 °C at the default rate of 0.2 °C/s, and fluorescence data was collected continuously (all points) during the ramp. During the reaction, data were collected from two replicates, and the results were presented collectively.

**Table 1:** Sequences of LAMP primers for detecting *Enterococcus hirae* (target *sod*A gene)

Primers name	Sequence (5′-3′)	Base pair length
F3 (forward outer primer)	CCTACAGATATCAAGACTGCTG	22
B3 (backward outer primer)	GCTGTTGAAGTGATCGCTA	19
FIP (F1c + F2; forward inner primer)	ACCAGCATTTGGTGCCATGAGTAATAATGGTGGCGGACAT	40 (20 F1c, 20 F2)
BIP (B1c + B2; backward inner primer)	CGAACCAACTGGTGCAATTAAAGAAAATTCTTCCTTAAATGTTGCAAAATC	51 (25 B1c, 26 B2)
LoopF (loop forward primer)	TTCCAGAAGAAAGAATGGTTTGC	23
LoopB (loop reverse primer)	GCGATTGATGAAACCTTTGGT	21

FIP consists of the F1c and F2 sequences; BIP consists of the B1c and B2 sequences. F1c and B1c were shown as underlined nucleotide sequences

Amplicon size amplified with the outer primers F3/B3 is 248 bp (Novazym Poland s.c., Poznań)

Reaction time was assessed with and without loop primers to determine their influence on the speed of the LAMP assay. To confirm reaction accuracy, LAMP products were detected by separation in 2% agarose gel with ethidium bromide staining and were visualized under UV light (UVP, US). The presence of specific multiple ladder bands was considered a positive result. Finally, the LAMP product of *E. hirae* ATCC 10541 was confirmed by sequencing analysis. After gel electrophoresis, the shortest bands on agarose gel were cut out, purified with the GeneMATRIX Agarose-Out DNA Purification Kit (EURx, Gdańsk, Poland) and sequenced by Genomed (Warsaw, Poland). The obtained sequence fragments were analyzed using the NCBI BLAST Sequence Analysis Tool (https://blast.ncbi.nlm.nih.gov/Blast.cgi).

### Specificity of the LAMP assay

The specificity of the LAMP assay was evaluated using the DNA of 7 *Enterococcus* reference ATCC strains: *E. casseliflavus* 700,327, *E. cecorum* 43,198, *E. durans* 6056, *E. faecalis* 29,212, *E. faecium* 700,221, *E. gallinarum* ATCC 700425 and *E. raffinosus* ATCC 49464), as well as 3 non-*E. hirae* strains (*E. coli* 25,922, *Staphylococcus aureus* 25,923, *Rimerella anatipestifer*). *E. hirae* ATCC 10541 was used as positive control. Nuclease-free water was used as negative control (NTC-Non Template Control).

### Standard curve and sensitivity of the LAMP assay

Two calibration methods were used to generate the standard curve. In the first method, quantification was based on the number of cells present in the bacterial suspension (colony-forming units, CFU) before DNA extraction. In the second method, bacteria were quantified in silico with the use of a calculator for determining the number of copies of a template (URI Genomics and Sequencing Center) available online at http://cels.uri.edu/gsc/cndna.html [34]. The results were presented as genome equivalents (GE). The size of the *E. hirae* genome (~ 2.9 Mb) from the NCBI database was used in the calculations. According to the literature, *sod*A is probably a single copy gene [35]. The entire bacterial genomic DNA was extracted from 1.3 × 10^8^ CFU/ml of *E. hirae* ATCC 10541 with the Genomic Mini AX Bacteria kit (A&A Biotechnology, Gdynia, Poland). The counts (CFU) of *E. hirae* ATCC 10541 were determined by plating on Enterococcosel Agar plates (Graso, Poland). DNA concentration was measured in the Nanodrop 2000 system (Thermo Scientific, USA). The concentration of bacterial genomic DNA was converted to genome equivalents in silico. Subsequently, a 10-fold dilution series of the DNA extracted from 1.3 × 10^8^ CFU/ml to 1 CFU/ml was prepared. Each dilution with a volume of 1 μl was used in duplicate as a quantitative standard for *Enterococcus*. A standard curve was generated by plotting Cq values against the logarithmic values of bacterial counts (log_10_ CFU equivalent and GE). The sensitivity of the LAMP technique was assessed with 10-fold serial dilutions (10^0^ to 10^− 8^) of *E. hirae* ATCC 10541 DNA with a concentration of 43.4 ng/μl up to 0.434 fg/μl, which corresponded to 0.138 to 1.38 × 10^7^ genome copies per reaction.

### The sensitivity of LAMP vs. PCR

The sensitivity of the LAMP technique and a standard PCR assay was compared with the use of the same DNA templates with identical concentrations and volumes. Two standard PCR assays were performed. The first assay involved outer primers F3/B3 (amplicon size 248 bp) from the LAMP test conducted in this study, and the second assay involved *E. hirae*-specific primers (amplicon size 187 bp) described by Jackson et al. [23]. Both PCR assays were performed in a volume of 25 μl containing 12.5 μl of the DreamTaq™ Green PCR Master Mix (2x) mix (K1081, Thermo Fisher Scientific Inc., USA)., 0.5 μl of each primer (10 pmol/μl), 1 μl of DNA and 10.5 μl of water. The amplification profile was as follows: initial denaturation at 95 °C for 4 min, followed by 30 cycles of 95 °C for 30 s, 55 °C for 60 s, 72 °C for 60 s, and final extension at 72 °C for 7 min [23]. The amplified DNA fragments were analyzed by electrophoresis in 1.2% (*w*/*v*) agarose gel. The PCR products obtained with LAMP primers (F3/B3) for *E. hirae* ATCC 10541 and randomly selected heart samples were verified by sequencing.

### Detection of *E. hirae* in hearts by the standard plate count (SPC) method

The results of the LAMP assay and conventional agar plate enumeration were compared based on *E. hirae* loads in the affected hearts. Heart samples were suspended in sterile water (ratio 1:10), and serial 10-fold dilutions were prepared in sterile water. Each dilution in the amount of 100 μl was plated on Enterococcosel Agar (Graso, Poland). Three replicate plates for each dilution were inoculated, and the colony counts on each plate were averaged. The number of colony-forming units per gram (CFU/g) of heart tissue was calculated using standard laboratory methods.

### Statistical methods

The Wilcoxon signed-rank test was used to determine statistically significant differences in *E. hirae* counts from LAMP and SPC assays. A two-tailed *p*-value of 0.05 was regarded as statistically significant. The agreement beyond chance between LAMP and PCR was assessed using Gwet’s AC1 coefficient rather than Cohen’s kappa to resolve the problem of unbalanced symmetrical marginal distribution of observations in the contingency table [36, 37]. Statistical analyses were performed in TIBCO Statistica 13.3 (TIBCO Statistics Inc.) and Microsoft Office Excel 2016.

## Results

**Fig. 3:**
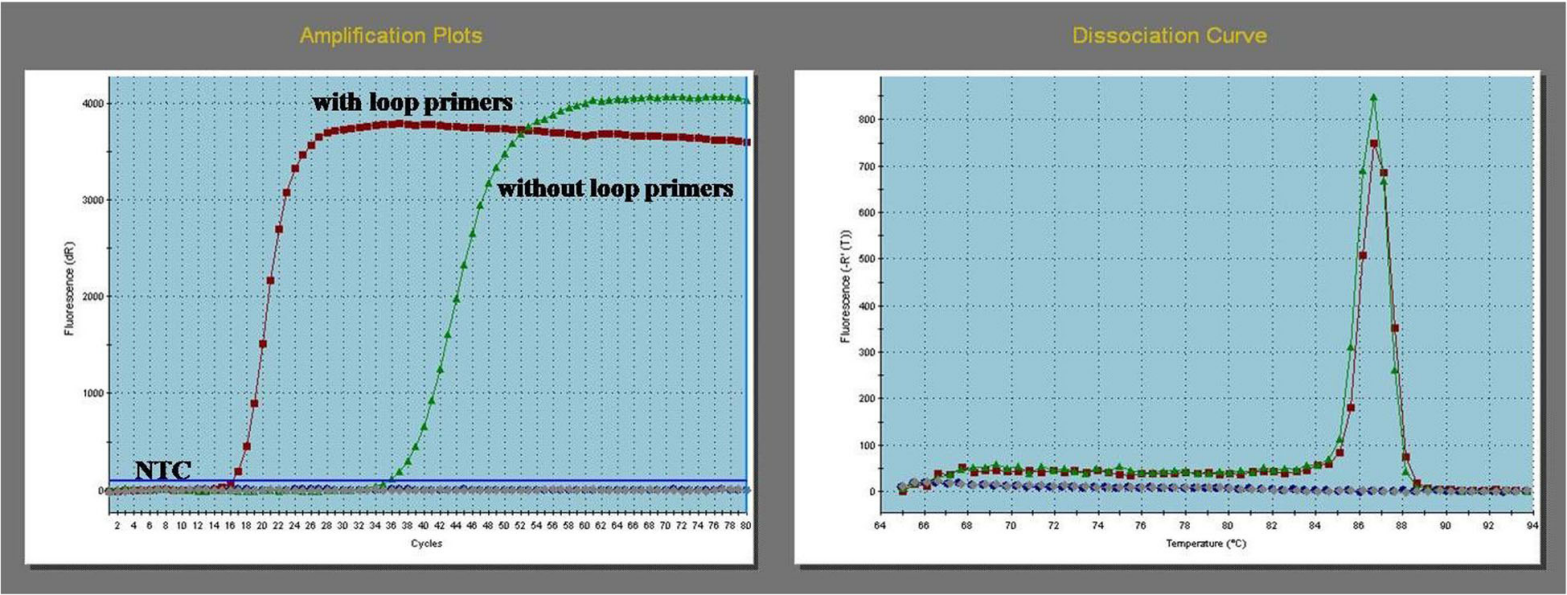
LAMP amplification graph and dissociation curves for *E. hirae* ATCC 10541 generated by running the assays with two and three sets of primers. Assay with the loop primers resulted in Cq value of 16.13, and melting peak at Tm 86.65 °C, whilst the one without the loop primers Cq value of 35.55, and melting peak at Tm 86.65 °C

The direct detection of LAMP products was based on melting temperature profiles determined by melting curve analysis of the amplified products. A single peak in the melting curve at Tm 86–87 °C was regarded as indicative of a set of pure, specific amplicons. The first amplified products of the *sod*A gene fragment from the reference strain of *E. hirae* were detected within 8 min with loop primers (Cq 16.13; Tm 86.65 °C), and within 18 min (Cq 35.55; Tm 86.65 °C) without loop primers (Fig. 3). The nucleotide sequence of the LAMP product of the control strain was highly similar (99%) to *E. hirae,* and it was deposited in GenBank (MG581167).

### Specificity of the LAMP assay

**Fig. 4:**
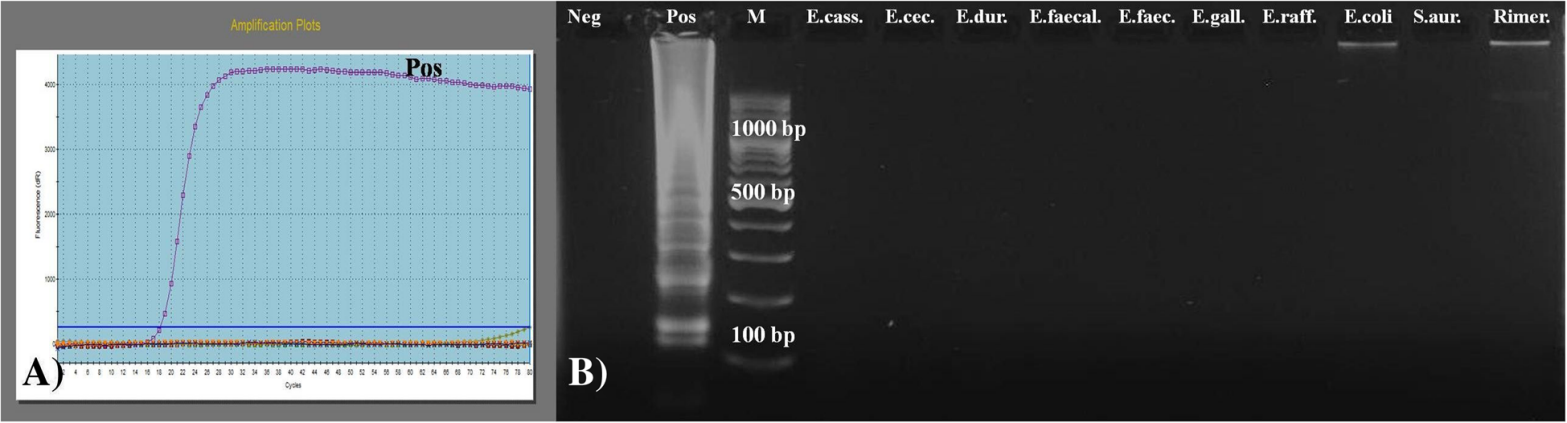
Amplification graph (**a**) and agarose gel (1.5*% w*/*v*) electrophoresis of LAMP products (**b**) amplified from genomic DNA of *E. hirae*, other *Enterococcus* and *non-Enterococcus strains*. Lane 1: Neg-negative control, Lane 2: Pos-positive control, *E. hirae* (ladder-like band pattern), Lane 3: M-100-bp DNA ladder (Thermo Fisher Scientific Inc., USA), Lane 4–13: *E. casseliflavus*, *E. cecorum*, *E. durans*, *E. faecalis*, *E. faecium*, *E. gallinarum*, *E. raffinosus*, *Escherichia coli*, *Staphylococcus aureus*, *Rimerella anatipestifer*

The LAMP assay demonstrated 100% specificity against different *Enterococcus* species and non-*Enterococcus* strains (Fig. 4a). Ladder-like DNA amplification products were detected only in *E. hirae* (positive LAMP reaction), and they were not identified in other strains (negative results) (Fig. 4b).

### The sensitivity of LAMP and conventional PCR assays

**Fig. 5:**
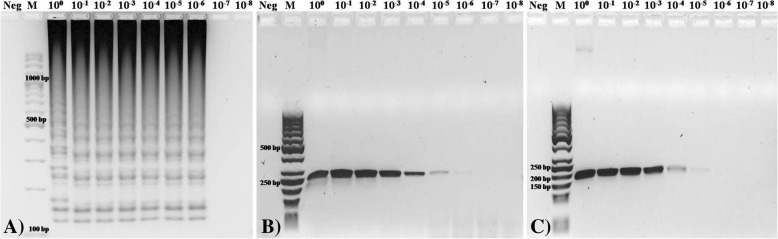
Agarose gel electrophoresis after (A) LAMP, and (B) PCR assay with F3/B3 LAMP primers and (C) species-specific primers using 10-fold dilutions of DNA *E. hirae* ATCC 10541 as a sensitivity indicator. **a**) Neg-Negative control, M-100-bp DNA ladder, Lane 3–11: LAMP assay products using serial dilutions **b**) Neg-Negative control, M-50-bp DNA ladder (SM0373,Thermo Fisher Scientific Inc., USA), Lane 3–11: PCR results using serial dilutions and F3/B3 LAMP primers (product length 248 bp) **c**) Neg-Negative control, M-50-bp DNA ladder, Lane 3–11: PCR results using serial dilutions and species-specific primers (product length 187 bp)

The sensitivity of the LAMP assay was tested using 10-fold serial dilutions of 1 μl of *E. hirae* ATCC 10541 DNA with a known number of colony counts and genome copies. Based on genome size and the concentration of genomic DNA, 43.4 ng of *E. hirae* ATCC 10541 DNA was equivalent to 1.38 × 10^7^ genome copies. The detection limit of the LAMP assay was determined at 1.3 × 10^2^ CFU (43.4 fg, 10^− 6^) or 13.8 copies of the *E. hirae* genome equivalent/reaction*,* and it was identical to that noted in the electrophoresis assay (Fig. 5a). The efficiency of the LAMP assay was 100%. The smallest detectable amount of *E*. *hirae* ATCC 10541 in the LAMP assay was obtained within 18 min, and the highest concentration was obtained within 8 min.

The detection limit of conventional PCR with F3/B3 primers was similar to that of the LAMP assay, but the band for the 10^− 6^ dilution appeared to be weak-positive (Fig. 5b). Conventional PCR with a set of *E. hirae*-specific primers had a detection limit of 1.3 × 10^3^ CFU (434 fg, 10^− 5^) or 138 genomic copies/reaction (Fig. 5c).

### The applicability of LAMP for analyses of *E. hirae* strains and heart samples

**Fig. 6:**
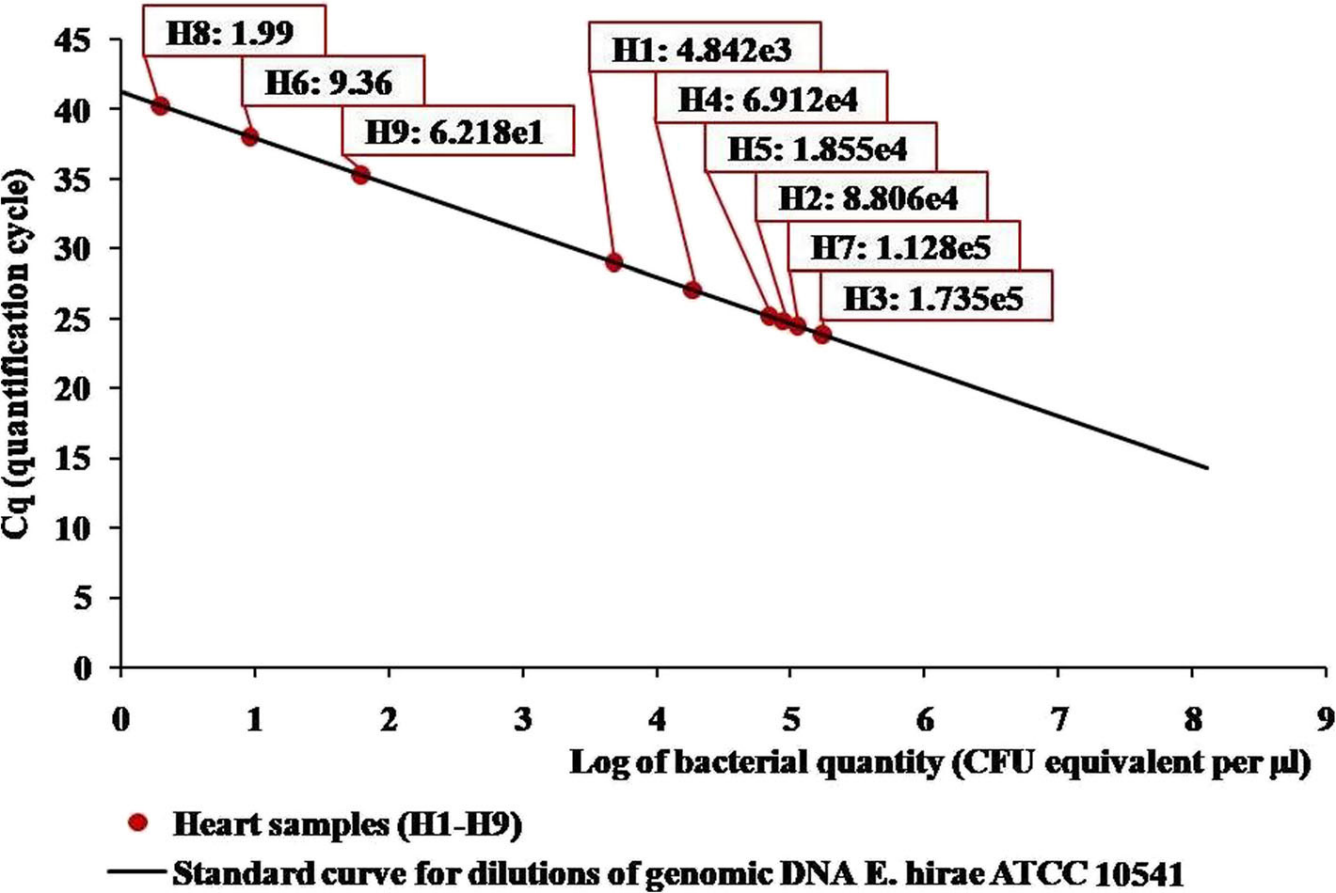
Results of LAMP assay for *E. hirae* load in affected chicken heart samples (log of CFU/μl of DNA template). Results for hearts were visualized on standard curve, which was generated from a dilution series of genomic DNA *E. hirae* ATCC 10541 by plotting the quantification cycle values (Cq) against the log of the bacterial quantity (log_10_ CFU equivalent per μl of DNA template). Linear equation: y = − 3.319x + 41.23. R^2^ = 0,99. Amplification factor = 2.00

LAMP products were detected in all of the tested 22 bacterial strains and all of the 9 heart tissue samples. The standard curve for LAMP-assisted quantification of bacterial DNA from hearts is presented in Fig. 6*.* Quantification results were also expressed in genome equivalents (GE) per gram of affected heart tissue (Table 2). In the LAMP assay, the average number of *E. hirae* equivalent genomes in hearts was determined at 5.51× 10^6^ copies/g.

**Table 2:** Detection of *E. hirae* in hearts of chickens representing different disease outbreaks

No. of affected heart	LAMP – cells/g	CFU counting	LAMP – GE/g
H3	1.74 × 10^8^	4.0 × 10^6^	1.84 × 10^7^
H7	1.13 × 10^8^	5.0 × 10^6^	1.2 × 10^7^
H2	8.81 × 10^7^	3.53 × 10^7^	9.35 × 10^6^
H4	6.91 × 10^7^	2.0 × 10^6^	7.34 × 10^6^
H5	1.86 × 10^7^	8.0 × 10^6^	1.97 × 10^6^
H1	4.84 × 10^6^	3.3 × 10^6^	5.14 × 10^5^
H9	6.22 × 10^4^	7.67 × 10^2^	6.6 × 10^3^
H6	9.36 × 10^3^	2.33 × 10^5^	9.94 × 10^2^
H8	1.99 × 10^3^	1.0 × 10^4^	2.12 × 10^2^
Mean	5.19 × 10^7^ *	6.43 × 10^6^ *	5.51 × 10^6^
SD	6.27 × 10^7^	1.11 × 10^7^	4.61 × 10^6^

LAMP – cells/g – bacterial cell number (log_10_ CFU equivalent) determined by LAMP

LAMP – GE/g – genome equivalents of *E. hirae* per g of heart sample calculated by LAMP

CFU – total viable *E. hirae* counts per g determined on selective agar after 24 h incubation

*statistical significance (*p* = 0.028) between bacterial cell number determined by LAMP and CFU (LAMP – cells/g vs. CFU counting)

The bacterial number of and genome equivalents were determined in 1 g of heart samples by LAMP and conventional plate-counting method

**Fig. 7:**
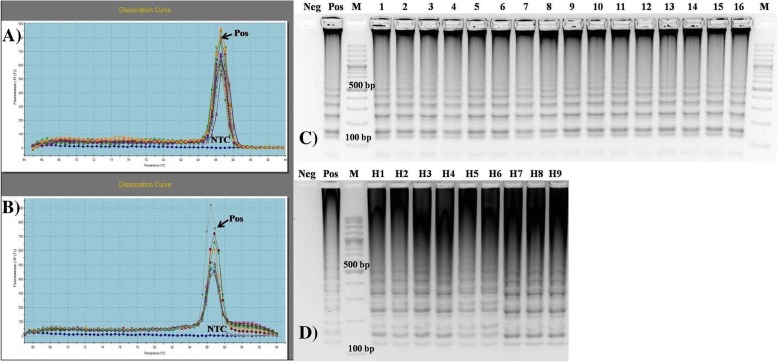
Melting curves generated after LAMP assay using (**a**) DNA of bacterial strains isolated from heart tissue samples and (**b**) DNA isolated directly from affected heart tissue samples of broiler chickens. Agarose gel electrophoresis of the LAMP products amplified from DNA of (**c**) bacterial strains isolated from heart tissue and (**d**) heart tissue samples of broiler chickens. Pos – positive control (*E. hirae* ATCC 10541), NTC - Non Template Control*,* Neg-Negative control, M-100-bp DNA ladder (SM0323, Thermo Fisher Scientific Inc., USA), **c**) Lane 4–19: bacterial isolates 1–16. **d**) Lane 4–12: 9 heart samples

**Fig. 8:**
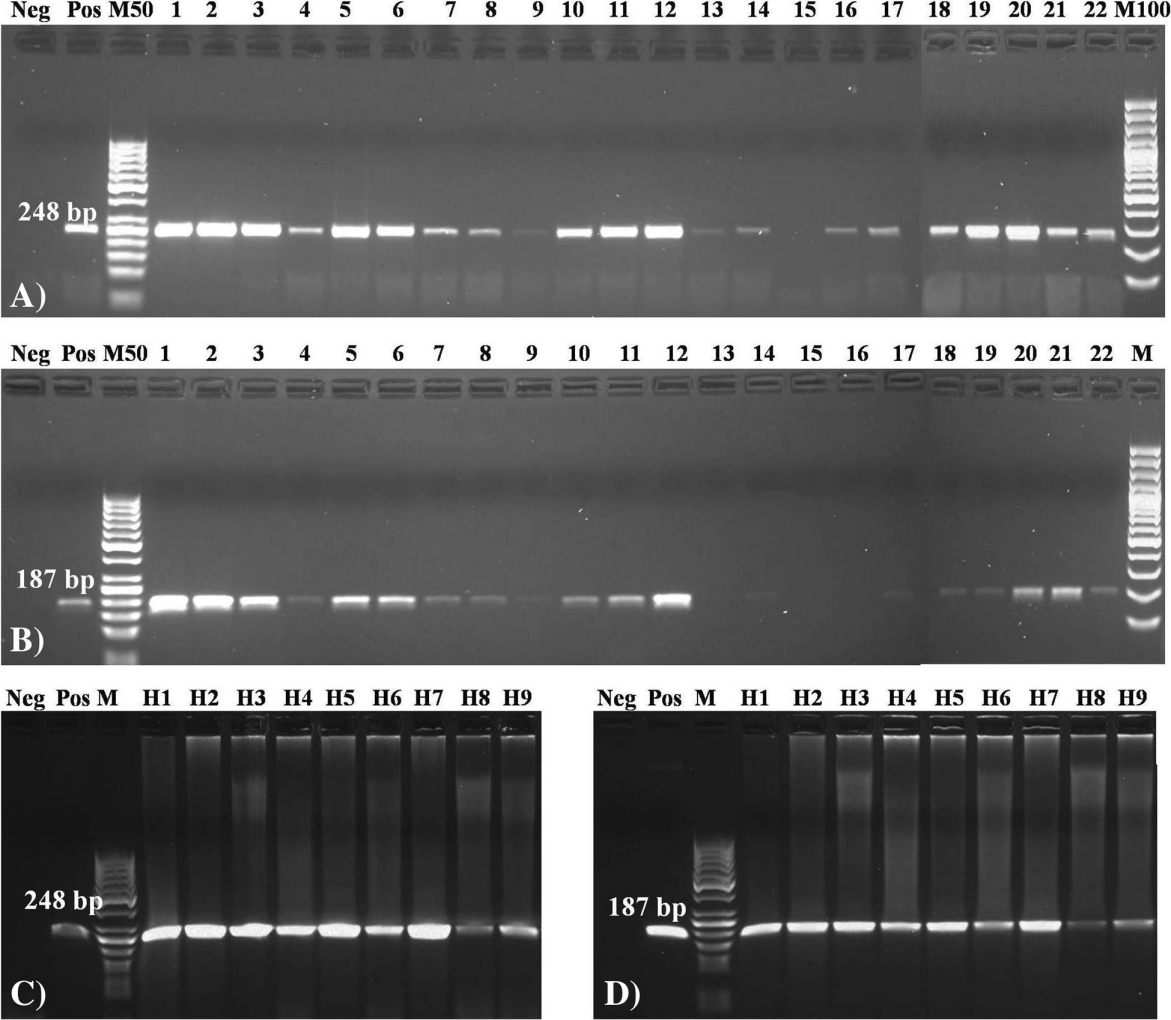
Agarose gel electrophoresis showing the PCR products generated using DNA of bacterial isolates and (**a**) F3/B3 LAMP primers or (**b**) species-specific primers, and showing the PCR products generated using DNA isolated directly from the heart tissue samples and (**c**) F3/B3 LAMP primers or (**d**) species-specific primers. Neg-Negative control, Pos-Positive control, M-50-bp or 100 bp DNA ladder (Thermo Fisher Scientific Inc., USA), **a**), **b**) Lane 3–11: 22 bacterial isolates. **c**), **d**) Lane 4–12: 9 heart tissue samples

Melting peaks were determined in the range of 86.05–87.06 °C (Fig. 7a) for field strains and in the expected range of 86–87 °C for hearts (Fig. 7b). Non-specific peaks were not detected for the tested primer sets. In bacterial strains and heart samples, the results of the LAMP assay were confirmed by the presence of a characteristic ladder*-*like pattern in agarose gel electrophoresis (Fig. 7c, d) and by sequencing. Standard PCR assays were less sensitive than LAMP. Twenty-one of the 22 analyzed isolates (95.5%) produced positive results in the PCR assay with F3/B3 primers, and one isolate (4.5%) was negative (Fig. 8a). Nineteen isolates (86.4%) produced positive results in PCR with species-specific primers, 2 isolates produced weak bands, and 3 isolates (13.6%) were negative (Fig. 8b).

The results of the LAMP test were consistent with the PCR results for heart samples obtained with LAMP primers and *E. hirae*-specific primers (Fig. 8c, d). The agreement beyond chance between LAMP and PCR is presented in Table 3. The newly generated sequence fragments from PCR with F3/B3 LAMP primers confirmed the high homology of *E. hirae* sequences. The obtained sequences were deposited in GenBank under accession numbers MG581168 (control strain) and MG581169 (heart sample). The fastest detection time of a LAMP product was approximately 7 min for *E. hirae* strains and 12 min for heart samples. The PCR assay was completed in approx. 2 h, and agarose gel electrophoresis was required to detect the amplified products.

**Table 3:** The agreement between LAMP assay and two standard PCRs (PCR with F3/B3 LAMP primers and PCR with species-specific primers)

Combination of tests	AC1
LAMP vs. PCR with F3/B3 LAMP primers^a^	95.4% (86.1–100%)
LAMP vs. PCR with species-specific primers^b^	84.4% (68.0–100%)
PCR with F3/B3 LAMP primers vs. PCR with species-specific primers	89.1% (74.7–100%)

AC1: the first-order agreement coefficient

^a^LAMP primers from this study

^b^Primers from Jackson et al. 2004

### Enumeration of *E. hirae* in heart samples by the standard plate count (SPC) method

All LAMP-positive heart samples were examined for bacterial counts on plate agar. The results of *E. hirae* quantification in heart samples by LAMP and culture assays are presented in Table 2.

## Discussion

Infections associated with enterococci often lead to growth depression and higher early mortality without specific clinical symptoms [1, 16]. *Enterococcus hirae* is not the only cause of endocarditis in chickens, and other pathogenic agents include *Enterococcus faecalis*, *E. faecium*, *E. durans*, *Staphylococcus aureus*, *Staphylococcus simulans*, *Streptococcus gallinaceus*, *Streptococcus pluranimalium*, *Avibacterium endocarditidis*, *Gallibacterium anatis* and *Helcococcus ovis* [38–43]. For this reason, species-specific methods are needed for rapid and accurate identification of the causative agent. In this study, the LAMP method was used to diagnose *Enterococcus hirae* infections in poultry. Other authors relied on the LAMP technique to detect bacterial pathogens such as *Salmonella spp., Streptococcus spp., Mycoplasma synoviae*, *Riemerella anatipestifer* and *Gallibacterium anatis* in poultry [29, 30, 42, 44, 45]. A review of the literature revealed that the previously described LAMP assays for *Enterococcus* spp. had been developed primarily to target enterococci in water [46, 47]. Kato et al. [48] demonstrated that LAMP was a useful technique for the rapid detection of *E. faecalis* and for diagnosing persistent endodontic infections. In the LAMP assays for detecting bacteria responsible for endocarditis, bacteria from the samples had to be previously cultured and DNA had to be isolated from a pure culture [49, 50]. In other studies, bacteria (including the causative agents of endocarditis, but not *E. hirae*) were used directly in the LAMP test without prior culturing [48, 51, 52]. In this study, *E. hirae* were detected in clinical isolates and heart tissue samples for the first time with the use of the LAMP assay. The melting temperature of specific amplicons was determined at 86–87 °C. Positive reactions were further confirmed by agarose gel electrophoresis and sequencing. The specificity of the LAMP assay for *E. hirae* was confirmed in a reaction with DNA samples from other enterococcal and non-enterococcal species.

Loop primers were applied to shorten reaction time. Nagamine et al. [25] demonstrated that loop primers accelerated LAMP. In our study, the LAMP assay with loop primers supported product detection in the first 10 min of amplification. In LAMP assays without loop primers, the product was detected around 10 min later. Similarly to our studies, the highest available concentration of bacterial DNA in LAMP with loop primers was determined within approximately 10 min [42]. In the LAMP assay, the *sod*A gene was amplified within up to 60 min. The LAMP test was clearly faster than conventional PCR. Similar observations were made in a LAMP assay for the rapid detection of *E. faecalis* [48].

The detection limit of the LAMP assay was 1.3 × 10^2^ CFU, which indicates that LAMP was an effective technique for the detection and quantification of *E. hirae* in samples. Based on a review of the literature, Martzy et al. [47] concluded that the LAMP method can reliably detect 130 copies of *E. faecalis* target DNA per reaction within 45 min. In our study, the detection limit (43.4 fg) was approximately twice lower than that reported by Kato et al. [48] for *E. faecalis* (100 fg/tube). Taking into account the concentration (ng/μl) and volume (1 μl/reaction) of the reference DNA in the LAMP assay in this study, the detection limit was lower than that noted for *G. anatis* strains in LAMP and qPCR tests [42].

According to the literature, the bacterial sensitivity of the LAMP assay can be 10- to 1000-fold higher relative to PCR, and the LAMP test can be equally or more sensitive than real-time PCR [46, 47, 53–55]. In this study, the sensitivity parameters of LAMP and conventional PCR were compared in 10-fold serial dilutions of *E. hirae* ATCC 10541 template DNA. The sensitivity of the LAMP assay was comparable to that of conventional PCR with F3/B3 LAMP primers only, but agarose gel electrophoresis revealed that the minimum detectable dilution (10^− 6^) produced a barely visible band in the PCR assay. The sensitivity of the LAMP test for *E. hirae* was 10-fold higher relative to PCR with species-specific primers. *E. hirae* DNA was detected in heart samples with a generally small number of copies. PCR with F3/B3 primers and species-specific primers confirmed that LAMP amplified the correct target and was a highly specific technique for target amplification. These observations indicate that LAMP is a more effective method than PCR for detecting *E. hirae* in samples where lower bacterial counts are expected. Unlike standard PCR (with LAMP primers or species-specific primers), LAMP produced positive results for all *E. hirae* isolates and heart samples affected by *E. hirae*. The agreement beyond chance between LAMP and PCR was very high (> 80%), which indicates that these techniques can be used interchangeably. However, our results should be interpreted with caution due to the very small number of negative samples screened in each test. Further large-scale research is required to fully substantiate these results.

In an experimental challenge study, Chadfield et al. [56] induced endocarditis in intravenously inoculated 4-week-old chickens by administering approximately 10^8^ CFU of a clinical strain of *E. hirae*. The inoculation with *E. hirae* via brachial and jugular veins produced culture-positive heart samples in 35 and 73% of the birds, respectively. Cardiac lesions included valvular endocarditis and were observed in 20 and 55% of the birds infected via brachial and jugular veins, respectively. However, bacterial counts in the affected tissues have not been investigated to date. In this study, the LAMP technique was used to estimate *E. hirae* loads in the hearts of commercial chickens which represented separate disease outbreaks. The variations in bacterial counts in hearts (10^3^ – 10^8^) could be attributed to different stages of infection in the examined flocks or differences in the pathogenic potential of *E. hirae* isolates. In this study, the bacterial load in the affected hearts determined by the LAMP method was compared with SPC results. The LAMP assay revealed significantly higher bacterial counts than SPC. The mean counts of viable *E. hirae* in hearts were approximately 8.1 times lower in SPC than in the LAMP test. Unlike SPC where only viable bacteria (replicating cells) are detected, the LAMP test and conventional qPCR amplify the DNA of both live and dead cells because DNA remains stable after bacterial death. The LAMP assay was essential for the accurate enumeration of *E. hirae* in tissue specimens. However, the main limitation of the LAMP test could be its inability to discriminate between live and dead cells. In this study, the lowest *E. hirae* counts determined in hearts by standard plating were 6 times higher than the detection limit of the LAMP test. Our results indicate that the LAMP assay could be helpful in overcoming certain limitations of conventional phenotypic procedures and plate-based enumeration for the detection of *E. hirae*.

Obviously, the applicability of the LAMP assay is limited to the detection, identification and quantification of *E. hirae*, and it cannot discriminate between clinical and commensal (normal) isolates. It is difficult to determine whether an isolate is the cause of an infection or whether it is non-pathogenic and is detected in samples due to contamination. Each isolation should be correlated with the health status and clinical signs in poultry. Predisposing factors could be involved in the establishment of infection. This is in contrast to other studies where predisposing factors were not evident in chickens prior to infection [56]. The properties responsible for the pathogenic potential of clinical *E. hirae* strains have not been elucidated to date. The differences between normal and pathogenic *E. hirae* have not been identified either. Further research is needed to characterize clinical *E. hirae* isolates, especially in relation to the presence of virulence factors.

## Conclusions

To the best of our knowledge, this is the first report to describe the applicability of a *sod*A–based LAMP assay for the detection of *Enterococcus hirae*. It is also the first study to evaluate the applicability of the LAMP assay for the quantification of *E. hirae* in affected chicken heart samples. The LAMP method supported the identification and quantification of bacteria in DNA samples isolated directly from heart tissue without prior cultivation*.* The LAMP technique enabled fast, specific and sensitive quantification, and it can be applied as an alternative molecular diagnostic tool detecting *E. hirae* in veterinary samples. The LAMP assay may facilitate diagnosis of infective endocarditis in poultry, thus contributing to differential diagnosis and the selection of the appropriate treatment. In this study, the LAMP assay was successfully used to diagnose *E. hirae*-associated endocarditis in broiler chickens.

## Abbreviations

CB: Chicken broiler flocks (commercial broilers)
CFU: Colony-forming unit
GE: Genome equivalents
LAMP: Loop-Mediated Isothermal Amplification
NTC: Non-Template Control
SPC: Standard plate count method
Tm: Melting temperature
